# Supplementary material for: Structural basis of inhibition of human NaV1.8 by the tarantula venom peptide Protoxin-I
Source: Nat Commun. 2025 Feb 7;16:1459. doi: 10.1038/s41467-024-55764-z (PMC11805909; doi:10.1038/s41467-024-55764-z)
Supplement: Supplementary file 1 — Supplementary Information [file 41467_2024_55764_MOESM1_ESM.pdf]

Supplementary Information

Structural basis of inhibition of human $Na_V1.8$ by the tarantula venom peptide Protoxin-I

Bryan Neumann[1], Stephen McCarthy[1], Shane Gonen[1*]

[1] Department of Molecular Biology and Biochemistry, University of California, Irvine, Irvine, CA 92697, USA
* to whom correspondence should be addressed.

Shane Gonen: gonens@uci.edu

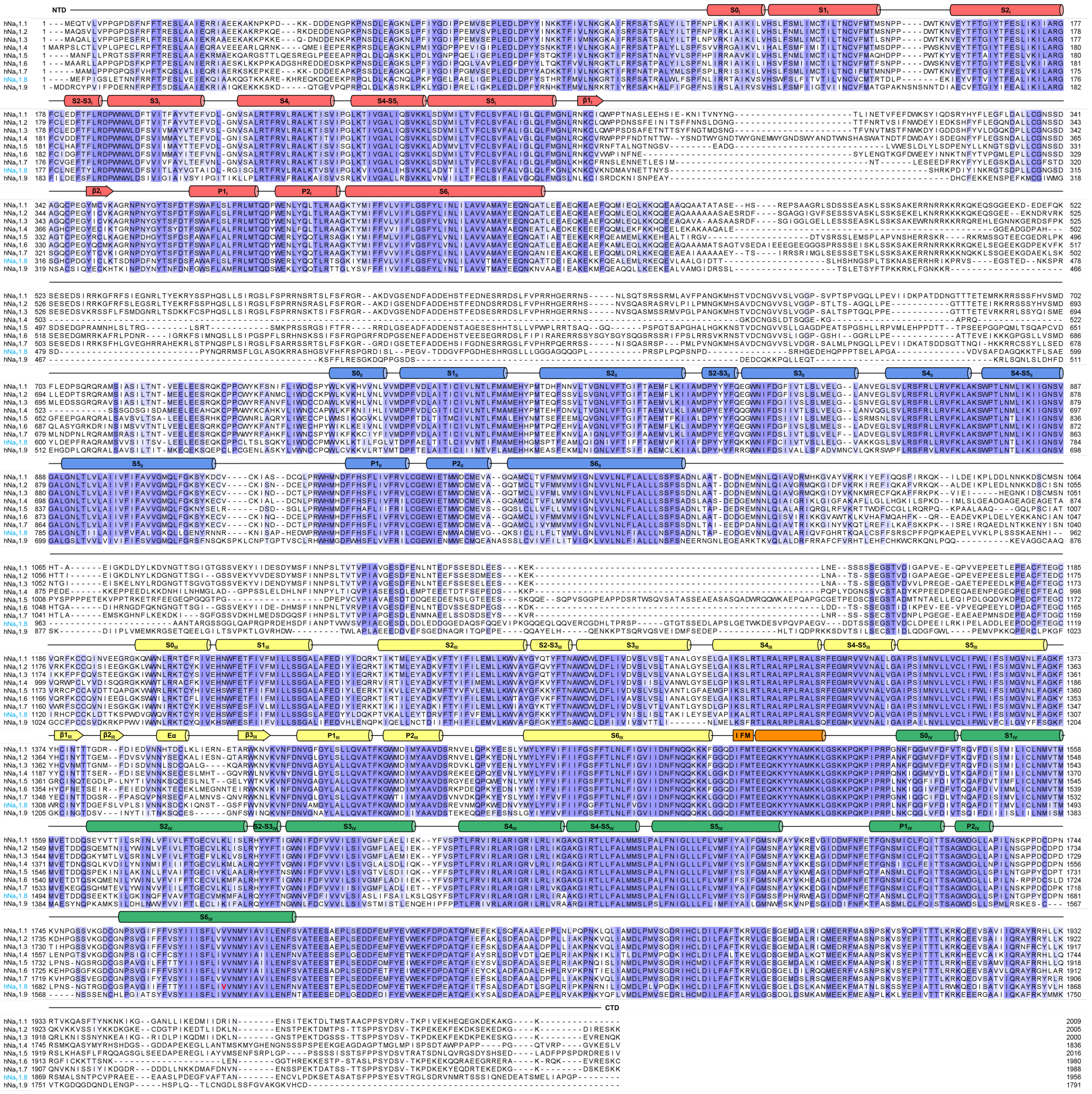

Supplementary Figure 1: Sequence alignment of hNa$_V$1.1-1.9

Sequences were aligned using ClustalΩ[1], and viewed in Jalview[2]. Residues are colored by conservation score with M1713V colored red; domains are colored following the scheme in Figure **1a**.

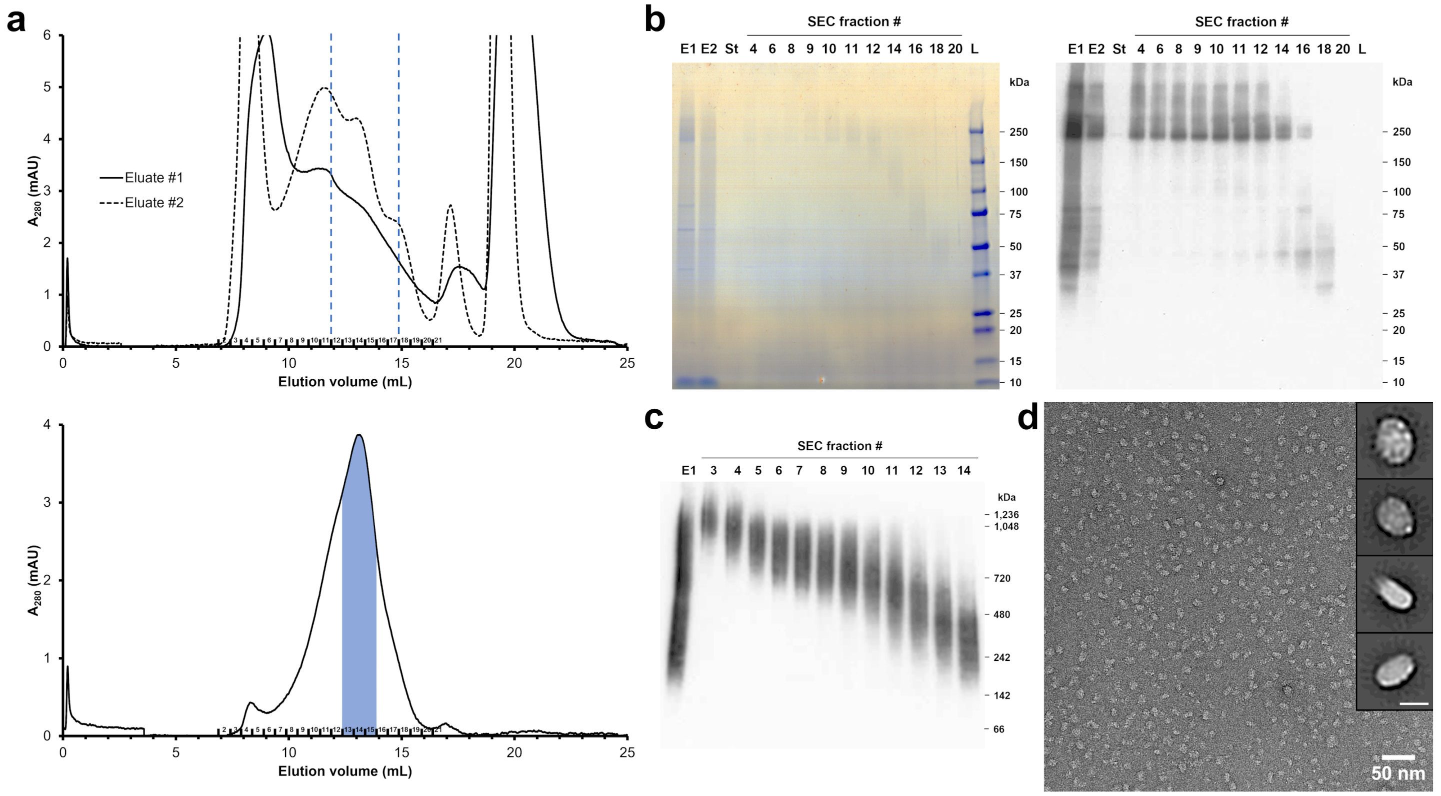

Supplementary Figure 2: Biochemistry and purification of apo-hNa$_V$1.8
a (upper) Example size-exclusion chromatography (SEC) traces following FLAG resin purification; the Eluate #1 trace shown as a solid black line and Eluate #2 as a dashed black line. Fractions between the dashed blue lines were pooled for (lower) subsequent SEC purification. Fractions highlighted in solid blue (F13-15) were carried forward for cryoEM. **b** SDS-PAGE gels of the fractions from the final SEC purification, with proteins detected by (left) Coomassie blue stain and (right) anti-FLAG western blotting. **c** Native PAGE gel of the fractions from the final SEC purification, with proteins detected by anti-FLAG western blotting. **d** Representative micrograph of negatively-stained particles from pooled fractions F13-15 with (inset) selected 2D class averages. Inset scale bar = 15 nm.

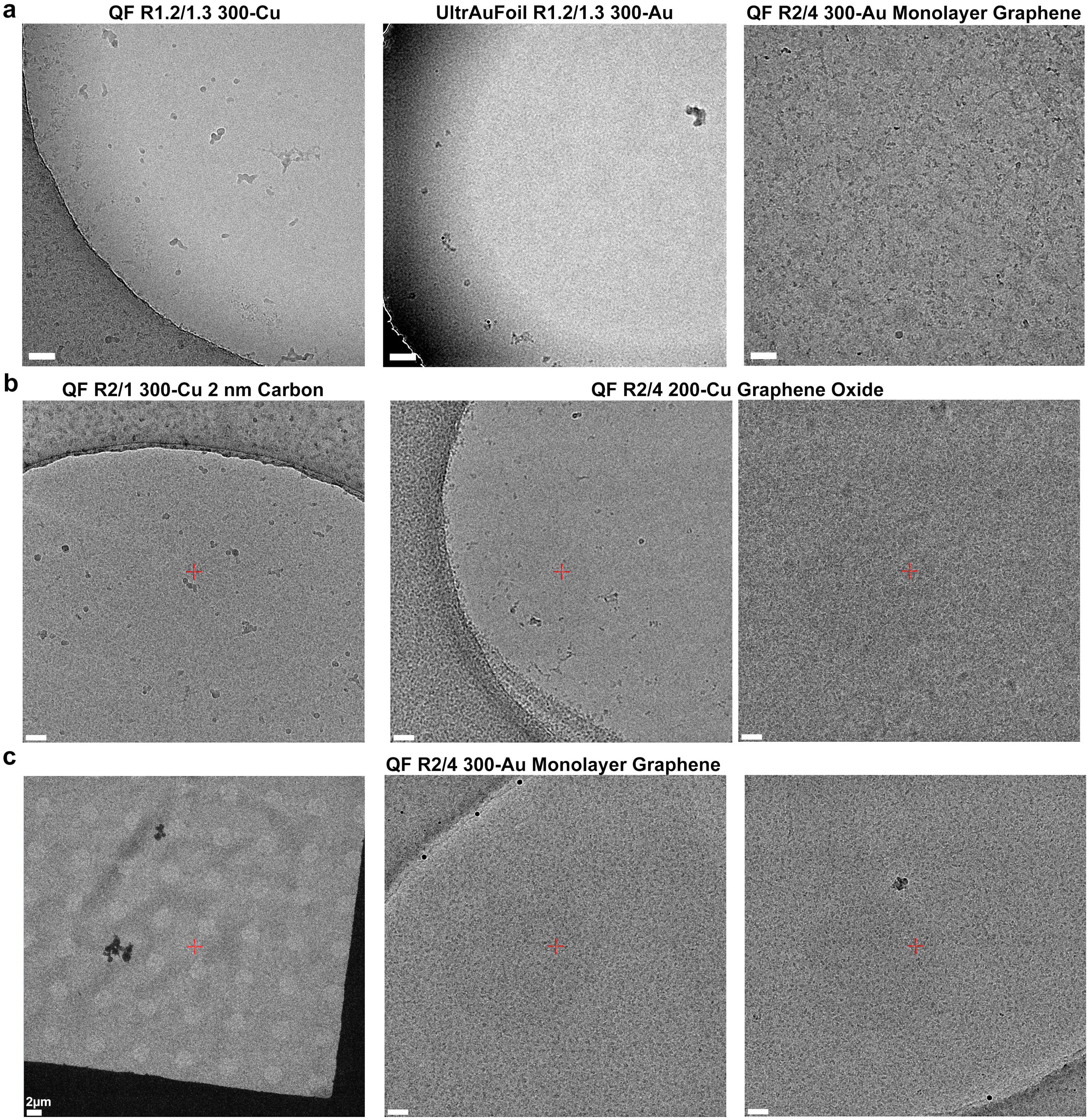

Supplementary Figure 3: Grid freezing overview outlining particle distribution on grids with and without support films

Representative cryoEM grid images of apo-hNa$_V$1.8. Scale bars are 50 nm unless otherwise indicated. **a** Example images of different grid types during initial screening; (left) Quantifoil R1.2/1.3 300-Cu grids show particles on carbon support rather than in holes; (center) UltrAuFoil R1.2/1.3 300-Au grids show particles in thick ice at the edge of the holes; and (right) Quantifoil R2/4 300-Au monolayer graphene grids show improved particle distribution. **b** Example images of different support film grids; (left) 2 nm carbon support film shows particles predominantly distributed on the ultrathin carbon support; (center) graphene oxide grids show frequent breakage after glow discharging even as (right) they show good particle distribution on the support film. **c** Example images of the grid leading to the apo-hNa$_V$1.8 reconstruction showing good particle distribution and contrast on the monolayer graphene support.

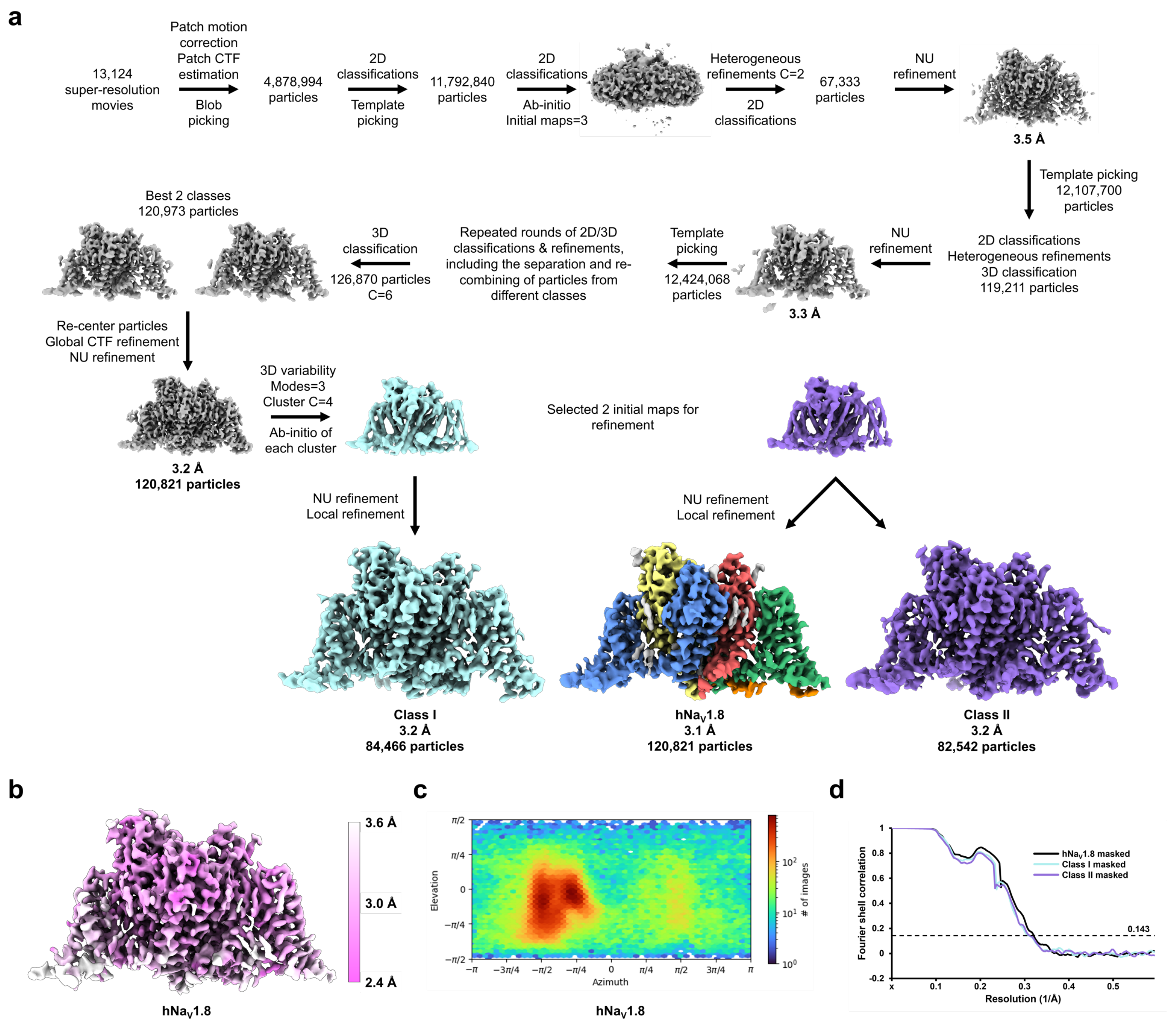

Supplementary Figure 4: Processing flowchart for apo-hNa$_V$1.8 using CryoSPARC

a Processing workflow resulting in the apo-hNa$_V$1.8 reconstruction, along with Class I and Class II. **b** Local resolution of apo-hNa$_V$1.8 calculated within CryoSPARC. **c** Angular distribution plot of particles in the final apo-hNa$_V$1.8 reconstruction. **d** Fourier shell correlation (FSC) plot of the three final apo-hNa$_V$1.8 reconstructions. FSC = 0.143 is indicated by a dashed black line.

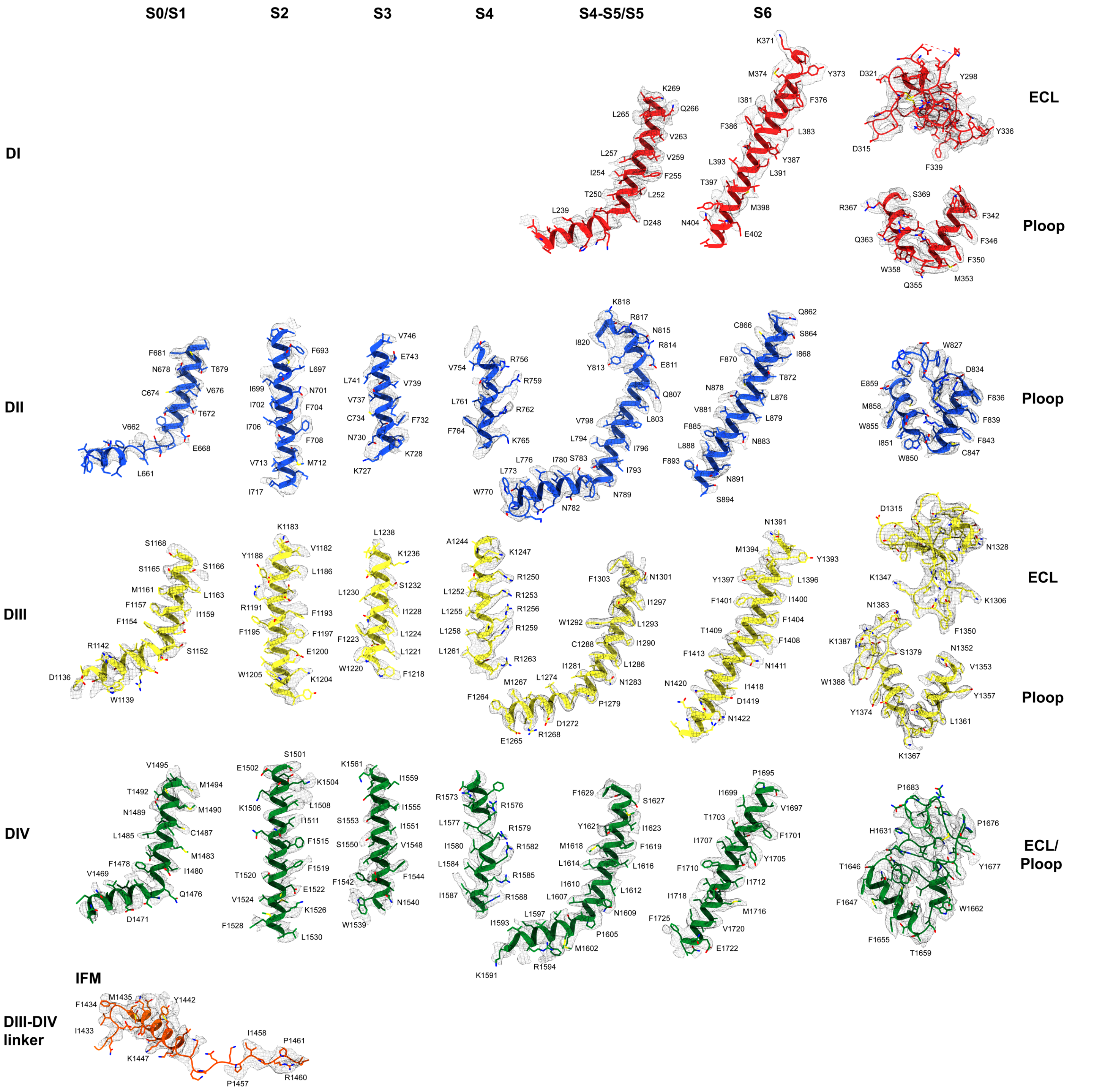

Supplementary Figure 5: apo-hNa$_V$1.8 model-to-map fit
All domains are colored according to the scheme in Figure **1a**.

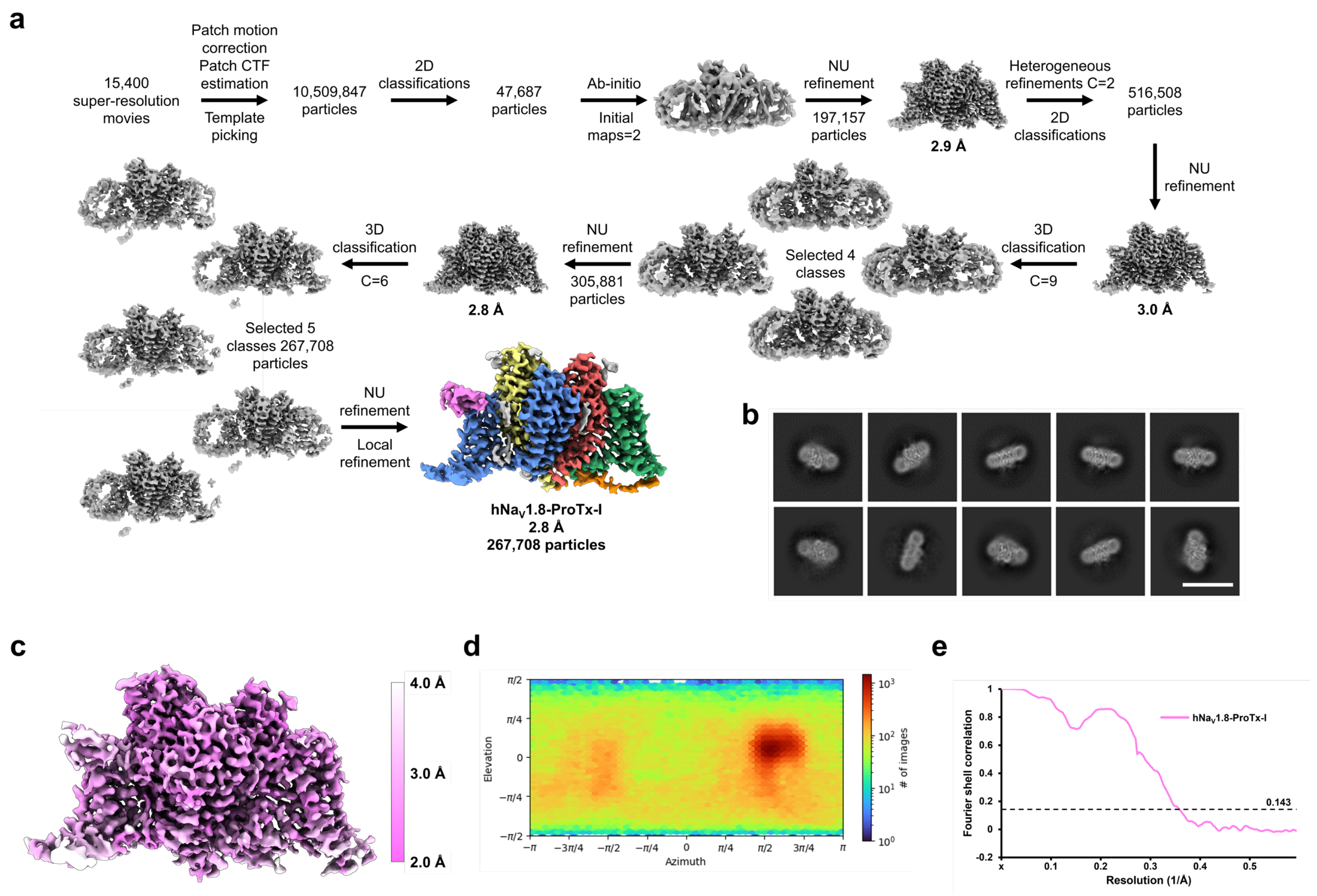

Supplementary Figure 6: Processing flowchart for the hNa$_V$1.8-ProTx-I complex using CryoSPARC
a Processing workflow resulting in the hNa$_V$1.8-ProTx-I reconstruction. **b** Example 2D class averages. Scale bar = 15 nm. **c** Local resolution of hNa$_V$1.8-ProTx-I calculated within CryoSPARC. **d** Angular distribution plot of particles in the final hNa$_V$1.8-ProTx-I reconstruction. **e** FSC plot of the final hNa$_V$1.8-ProTx-I reconstruction. FSC = 0.143 is indicated by a dashed black line.

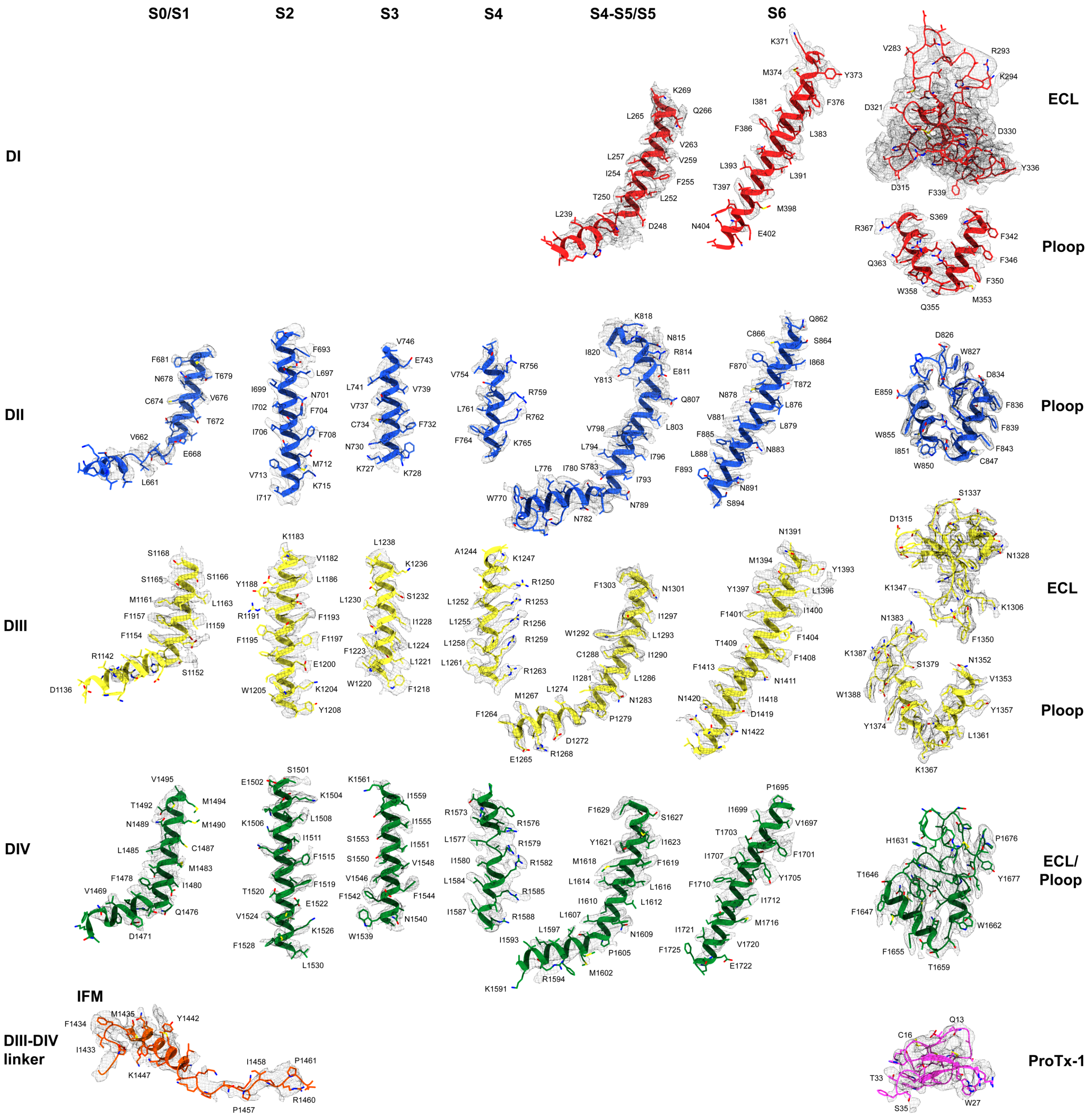

Supplementary Figure 7: hNa$_V$1.8-ProTx-I model-to-map fit
All domains are colored according to the scheme in Figure **1a**, with ProTx-I colored pink.

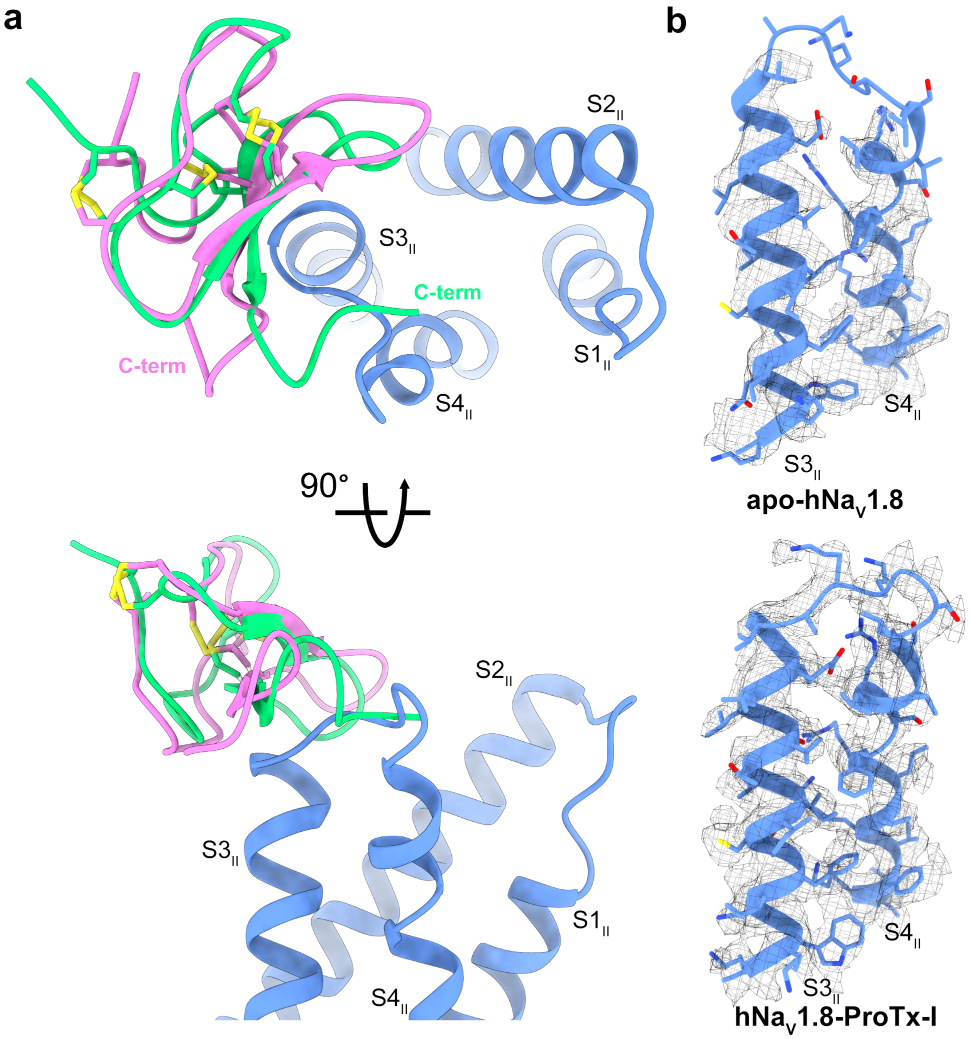

Supplementary Figure 8: ProTx-I interactions and alignment with hNa$_V$1.8
a Extracellular view of ProTx-I binding region highlighting the repositioning of the C-terminus. Bound ProTx-I from this study in pink and an NMR model of ProTx-I in green (PDB 2M9L). **b** Model-to-map fit of VSD$_{II}$ S3 and S4 helices for apo-hNa$_V$1.8 (top) and hNa$_V$1.8-ProTx-I (bottom) highlighting the observed comparative lower-resolution of the S3-S4 linker in apo-hNa$_V$1.8.

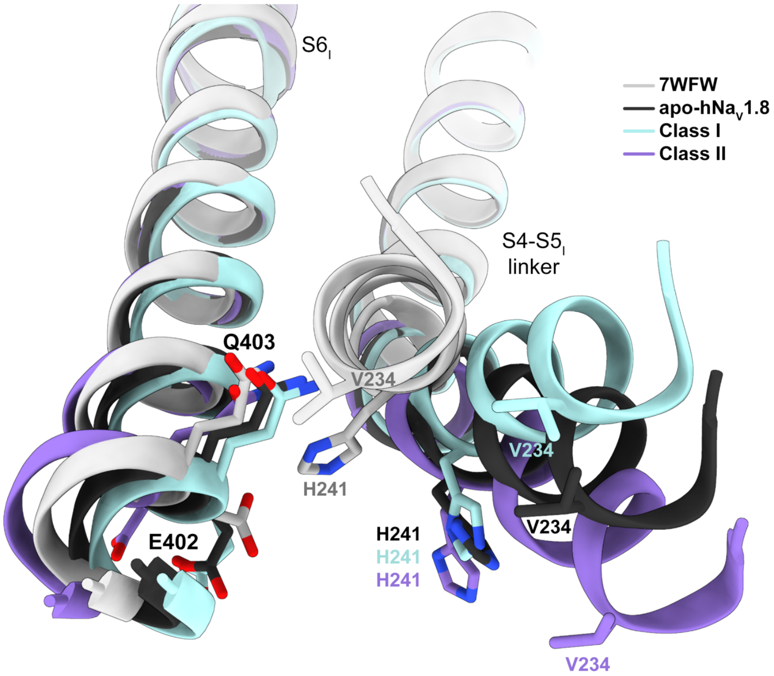

Supplementary Figure 9: DI S4-S5 linker and S6 region depicting location of key residues
Residues H241 and V234, unique to $hNa_V1.8$ are indicated, along with conserved residues E402 and Q403 on DI S6 helix. Domains are colored following the scheme in Figure **2a**.

	apo-hNa$_V$1.8	Class I	Class II	hNa$_V$1.8-ProTx-I
**Data collection**				
Microscope	Titan Krios			
Voltage (kV)	300			
Detector	Gatan K3			
Pixel size (Å)	0.839			0.827
Total electron dose ($e^-/Å^2$)	60			
Defocus range (µm)	–1 to –2.5			–1 to –2
# of movies	13,124			15,400
**Reconstruction**				
Software	CryoSPARC			
Symmetry	C1 (no symmetry)			
Selected movies	13,027			14,125
Final # of particles	120,821	84,466	82,542	267,708
Overall resolution (Å)	3.12	3.24	3.22	2.76
FSC threshold	0.143			
Map sharpening B factor ($Å^2$)	-73.97	-68.94	-64.86	-65.68
**Model Refinement**				
Model composition				
Non-hydrogen atoms	8,766	8,766	8,755	9,096
Protein residues	998	998	998	1047
Ligands	31	31	31	29
R.M.S. deviations				
Bond lengths (Å)	0.008	0.008	0.008	0.008
Bond angles (°)	0.928	0.981	1.014	1.151
Validation				
MolProbity score	1.73	1.86	1.81	2.09
Clashscore	9.15	9.99	10.51	12.76
Poor rotamers (%)	0.45	0.79	0.68	1.72
Ramachandran plot (%)				
Favored	96.36	95.15	96.06	95.77
Allowed	3.64	4.85	3.94	4.23
Outlier	0.00	0.00	0.00	0.00

Supplementary Table 1: Data collection parameters, model statistics and validation

Supplementary References

1. Sievers, F. *et al.* Fast, scalable generation of high-quality protein multiple sequence alignments using Clustal Omega. *Mol Syst Biol* **7**, 1–6 (2011).
2. Waterhouse, A. M., Procter, J. B., Martin, D. M. A., Clamp, M. & Barton, G. J. Jalview Version 2-A multiple sequence alignment editor and analysis workbench. *Bioinformatics* **25**, 1189–1191 (2009).
