# Supplementary material for: Structural basis of inhibition of human NaV1.8 by the tarantula venom peptide Protoxin-I
Source: Nat Commun. 2025 Feb 7;16:1459. doi: 10.1038/s41467-024-55764-z (PMC11805909; doi:10.1038/s41467-024-55764-z)
Supplement: Supplementary file 2 — Description of Additional Supplementary Files [file 41467_2024_55764_MOESM2_ESM.pdf]

Description of Additional Supplementary Files

File name: Supplementary Movie 1

Description: 3D variability analysis prior to final reconstruction of apo-hNaV1.8 highlighting the movements of the VSDI S4-S5 linker. Domains are colored following the scheme in Figure 1a.

File name: Supplementary Movie 2

Description: 3D variability analysis prior to final reconstruction of apo-hNaV1.8 highlighting the N-terminal domain (NTD). Domains are colored following the scheme in Figure 1a with the NTD density on the bottom left in white.

File name: Supplementary Movie 3

Description: Hydrophobic surface of ProTx-I from the hNaV1.8-ProTx-I complex. Colors follow the scheme in Figure 4a.

File name: Supplementary Movie 4

Description: Apo-hNaV1.8 to hNaV1.8-ProTx-I model morph highlighting the movement of the VSDII S3-S4 linker due to ProTx-I binding. Apo-hNaV1.8 is depicted in black and hNaV1.8-ProTx-I in blue with ProTx-I in pink.
