# Supplementary figures and images for: Structural basis of inhibition of human NaV1.8 by the tarantula venom peptide Protoxin-I
Source: Nat Commun. 2025 Feb 7;16:1459. doi: 10.1038/s41467-024-55764-z (PMC11805909; doi:10.1038/s41467-024-55764-z)

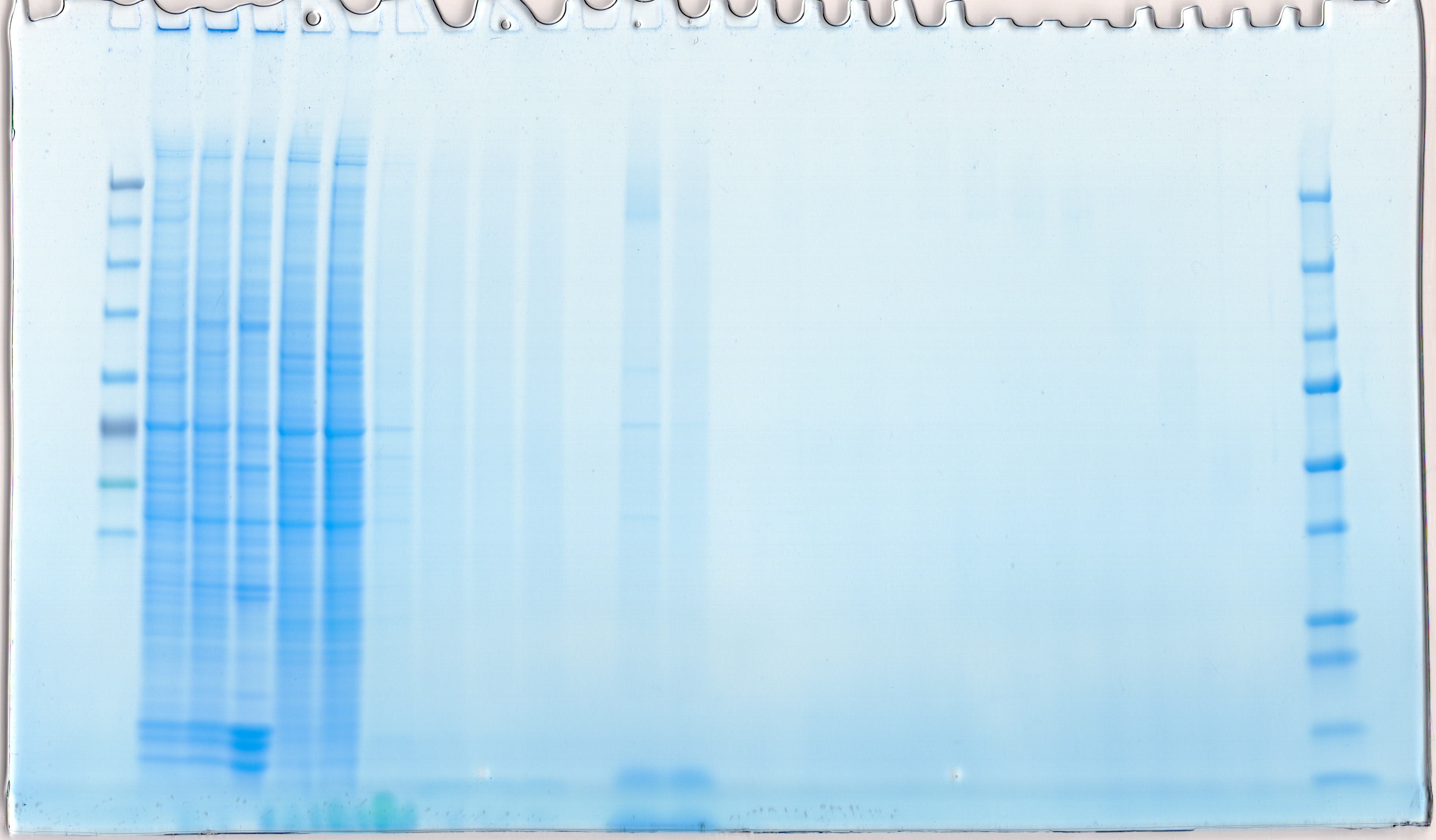

Supplement: Supplementary file 9 — Source Data [file 41467_2024_55764_MOESM9_ESM.zip › gels/coomassie.png]

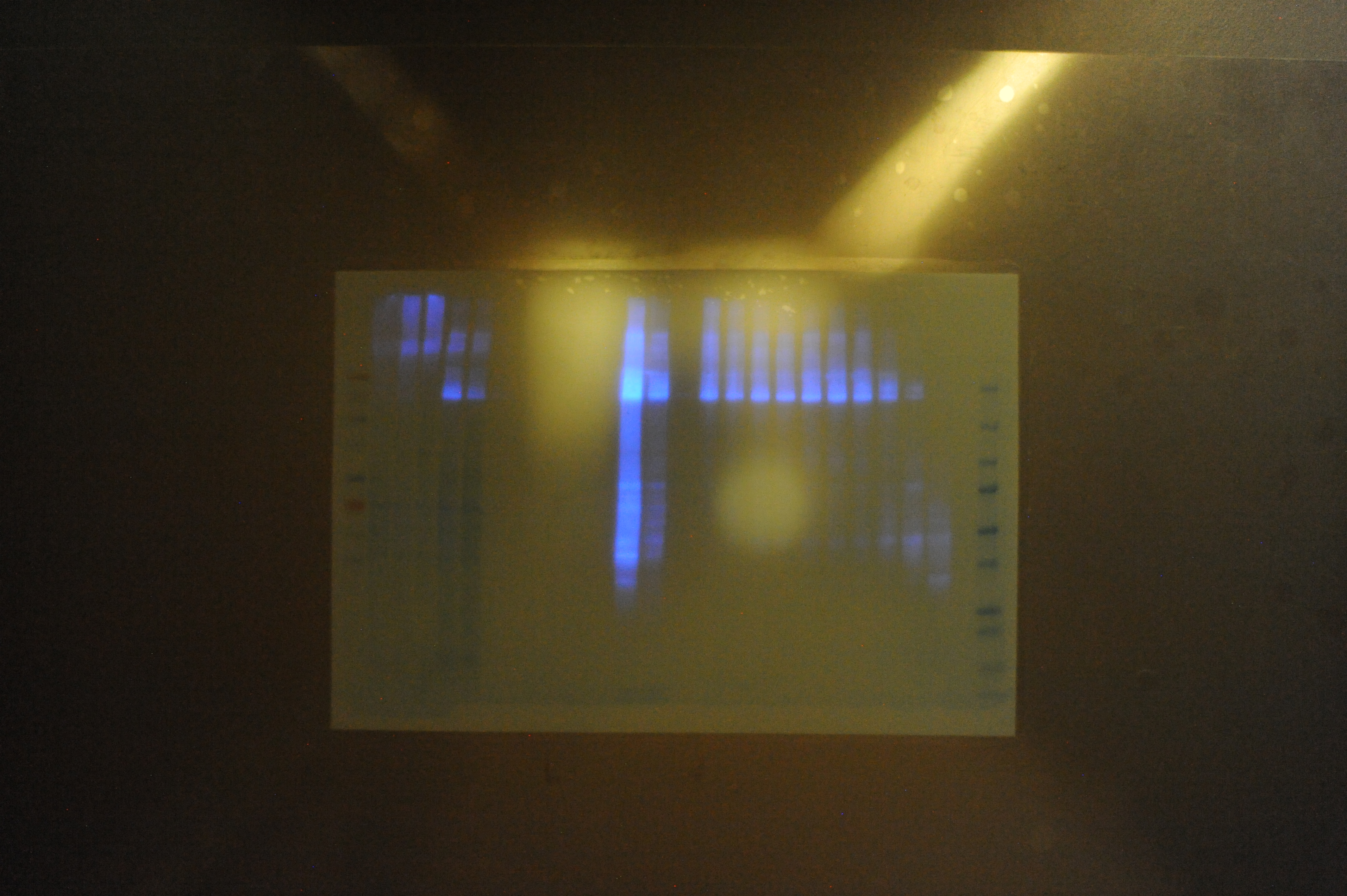

Supplement: Supplementary file 9 — Source Data [file 41467_2024_55764_MOESM9_ESM.zip › gels/sds visible.jpg]

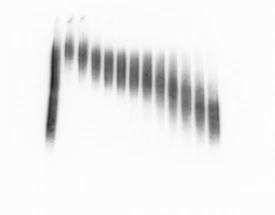

Supplement: Supplementary file 9 — Source Data [file 41467_2024_55764_MOESM9_ESM.zip › gels/native.png]

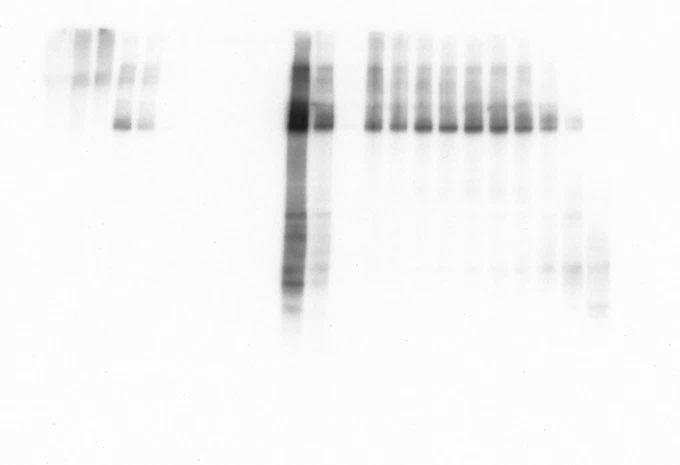

Supplement: Supplementary file 9 — Source Data [file 41467_2024_55764_MOESM9_ESM.zip › gels/sds.png]

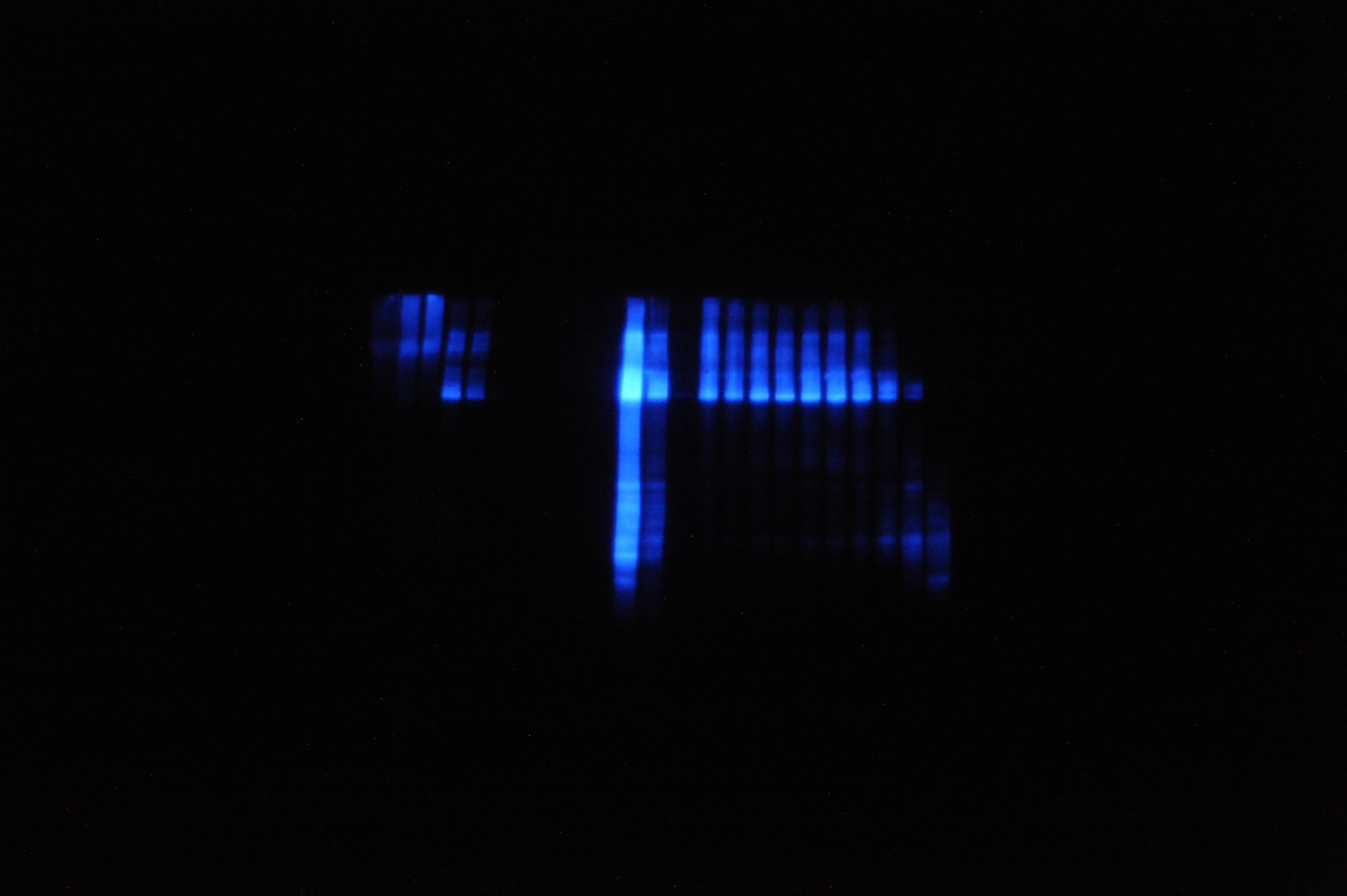

Supplement: Supplementary file 9 — Source Data [file 41467_2024_55764_MOESM9_ESM.zip › gels/sds 20s.jpg]

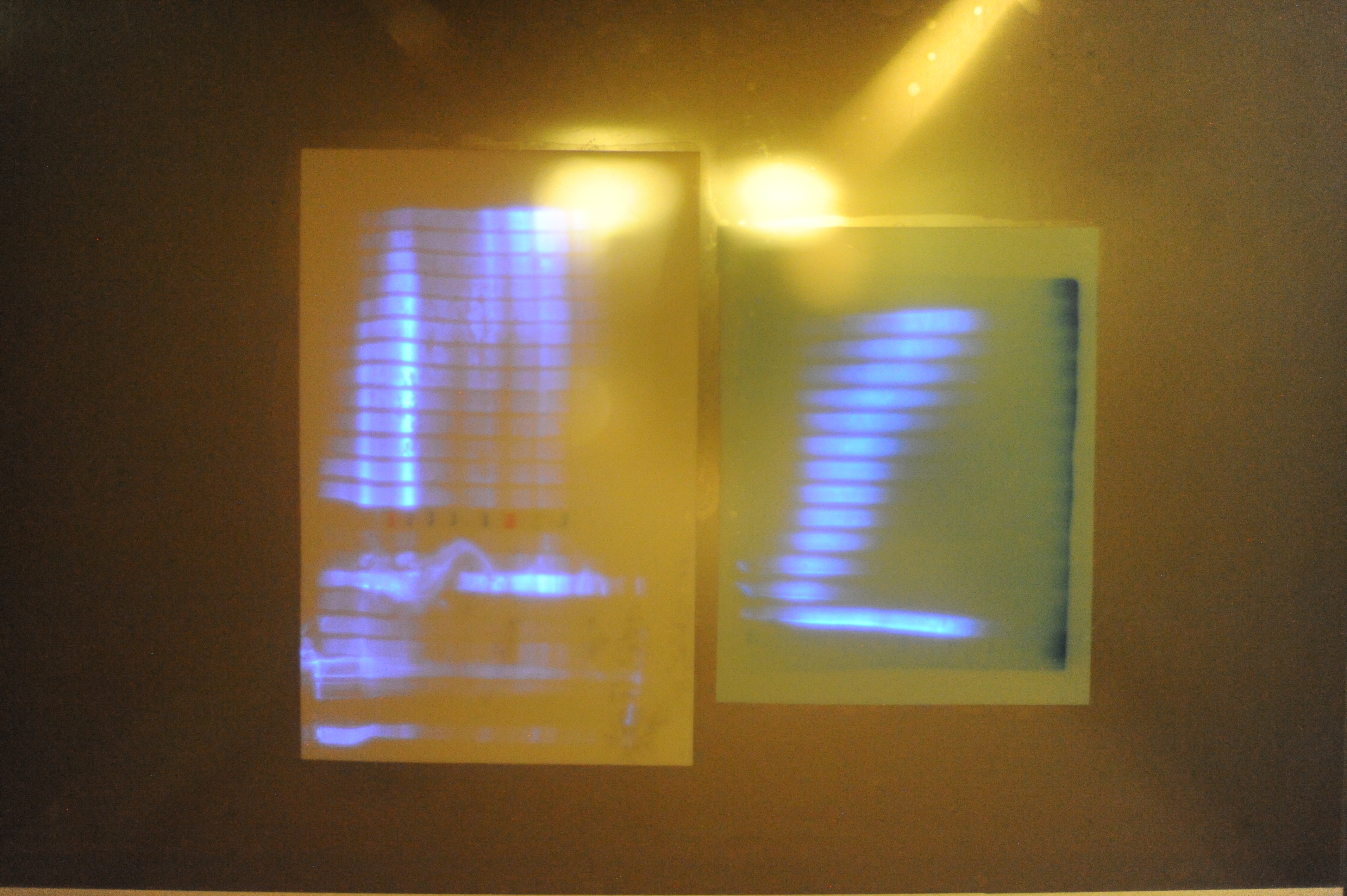

Supplement: Supplementary file 9 — Source Data [file 41467_2024_55764_MOESM9_ESM.zip › gels/native visible.jpg]

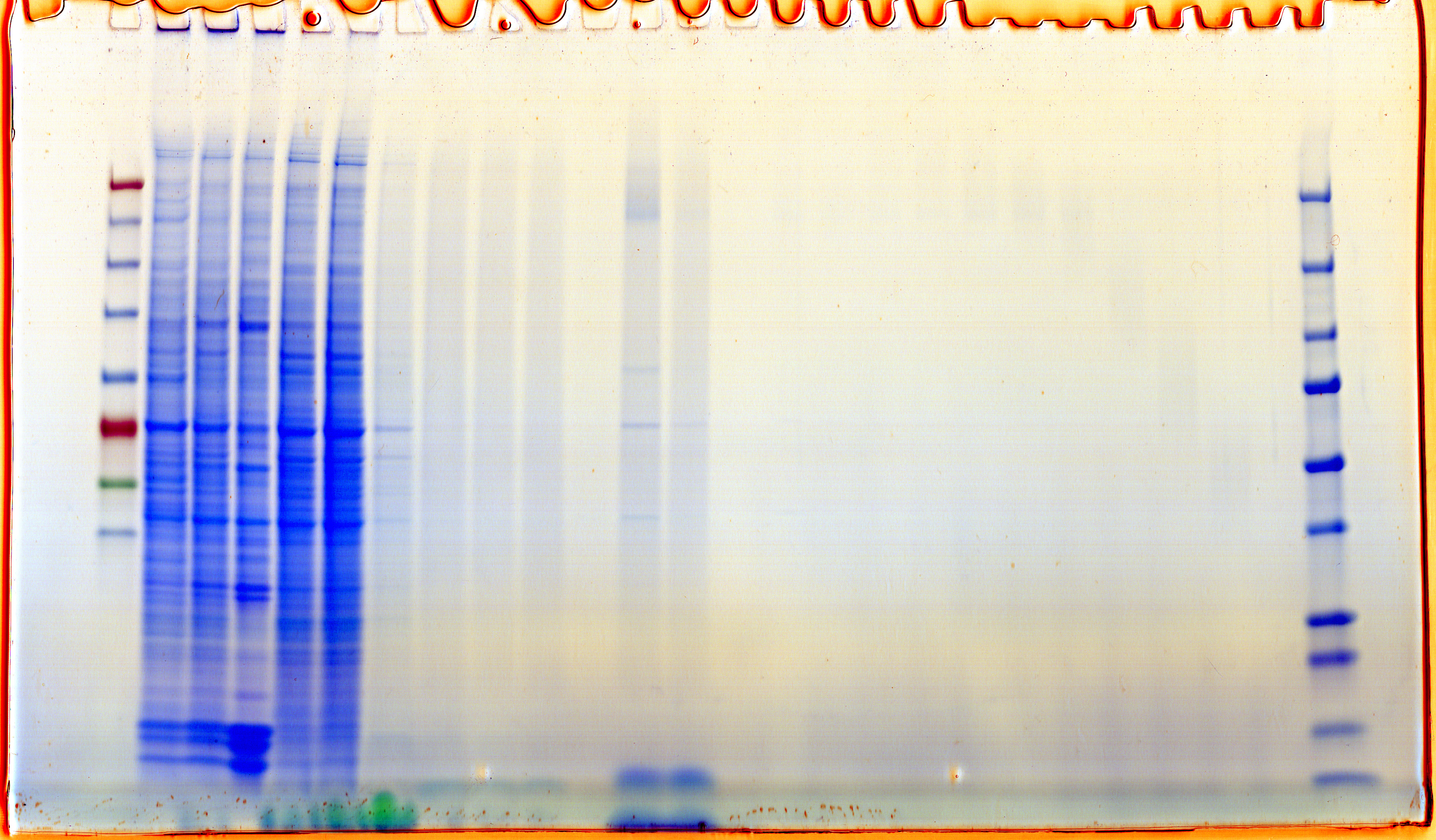

Supplement: Supplementary file 9 — Source Data [file 41467_2024_55764_MOESM9_ESM.zip › gels/coomassie_processed.png]

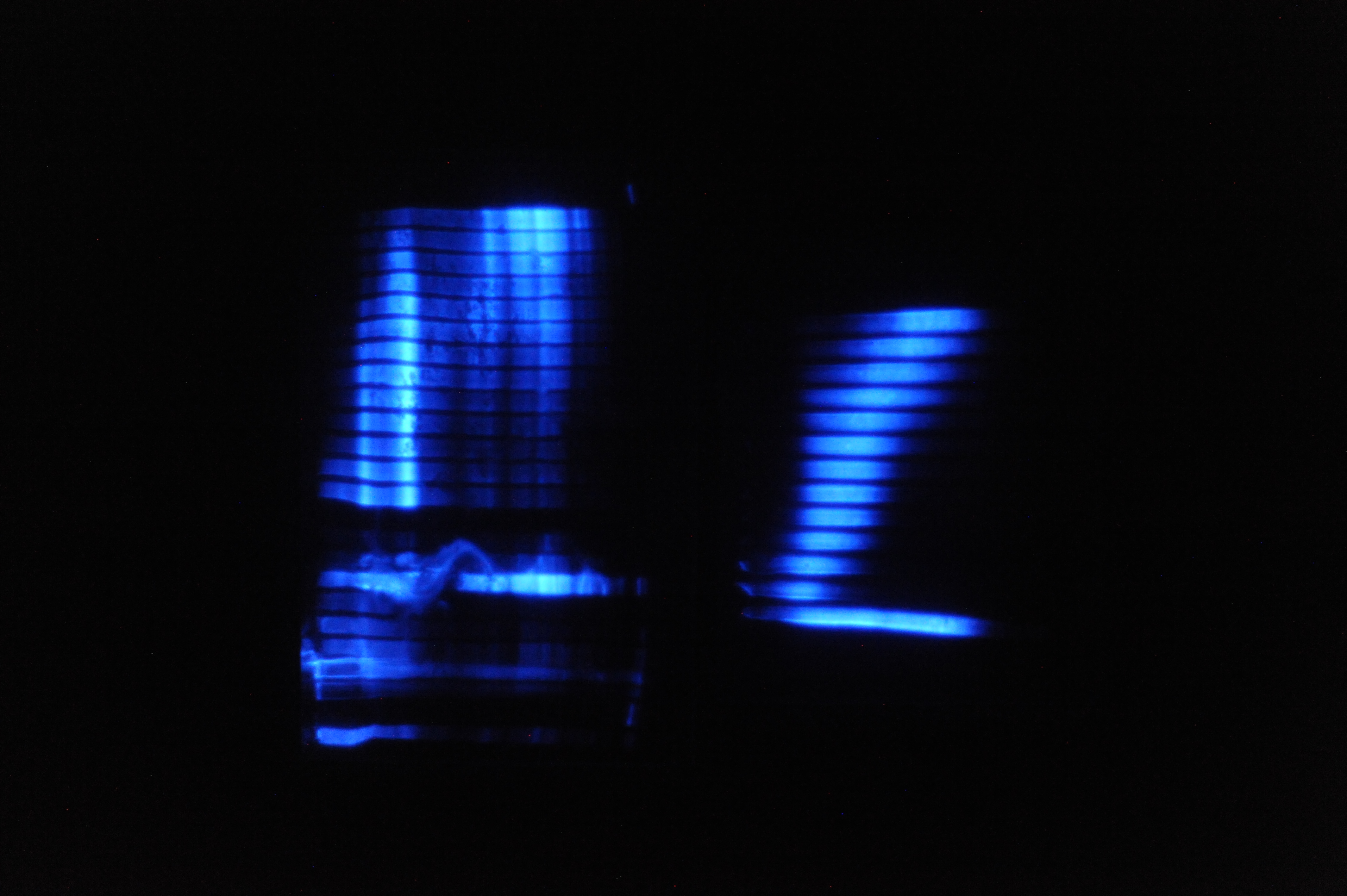

Supplement: Supplementary file 9 — Source Data [file 41467_2024_55764_MOESM9_ESM.zip › gels/native 10s.jpg]
